# A guide to selecting high-performing antibodies for Rab1A and Rab1B for use in Western Blot, immunoprecipitation and immunofluorescence

**DOI:** 10.12688/f1000research.143928.3

**Published:** 2025-03-07

**Authors:** Vera Ruíz Moleón, Charles Alende, Maryam Fotouhi, Riham Ayoubi, Sara González Bolívar, Kathleen Southern, Peter S. McPherson, Carl Laflamme, NeuroSGC/YCharOS/EDDU collaborative group, ABIF consortium

**Affiliations:** 1 Department of Neurology and Neurosurgery, Structural Genomics Consortium, The Montreal Neurological Institute, McGill University, Montreal, Québec, H3A 2B4, Canada

**Keywords:** Uniprot ID P62820 (Rab1A), Q9H0U4 (Rab1B), RAB1A and RAB1B, Rab1A, Rab1B, antibody characterization, antibody validation, Western Blot, immunoprecipitation, immunofluorescence

## Abstract

Rab1 is a highly conserved small GTPase that exists in humans as two isoforms: Rab1A and Rab1B, sharing 92% sequence identity. These proteins regulate vesicle trafficking between the endoplasmic reticulum (ER) and Golgi and within the Golgi stacks. Rab1A and Rab1B may be oncogenes, as they are frequently dysregulated in various human cancers. Moreover, they contribute to the progression of Parkinson’s disease. The availability of high-quality antibodies specific for Rab1A or Rab1B is essential to understand the distinct functions of these Rab1 proteins in both health and diseaseand to enhance the reproducibility of research involving these proteins. In this study, we characterized seven antibodies targeting Rab1A and five antibodies targeting Rab1B for Western Blot, immunoprecipitation, and immunofluorescence using a standardized experimental protocol based on comparing read-outs in knockout cell lines and isogenic parental controls. These studies are part of a much larger, collaborative initiative seeking to address the antibody reproducibility issue by characterizing commercially available antibodies for human proteins and publishing the results openly as a valuable resource for the scientific community. While uses of antibodies and protocols vary between laboratories, we encourage readers to use this report as a guide to select the most appropriate antibodies for their specific needs.

## Introduction

Multiple steps in membrane trafficking are coordinated by Rab proteins, a family of small guanosine triphosphatases (GTPase). ^1^ Rab GTPases undergo a dynamic cycle, alternating between an active GTP-bound state, catalyzed by guanine exchange factors (GEF), and an inactive GDP-bound state, achieved through GTP hydrolysis, stimulated by a GTPase-activating protein (GAP). ^1^^–^^3^ When activated, Rab proteins partake in crosstalk through shared effector proteins or through Rab activators to ensure vesicle traffic is spatiotemporally regulated. ^1^ Homologous to YTP1 in yeast, the Rab1 human proteins play key roles in regulating ER-Golgi and intra-Golgi transport. They exist as two isoforms in humans, Rab1A and Rab1B. While Rab1A and Rab1B share 92% amino acid identity, understanding their specific roles in membrane trafficking is a matter of ongoing investigation. ^4^^–^^6^ Rab1B has been proposed to function in the initial stages of the secretory pathways, serving to assemble and disassemble machinery required for vesicle fission and fusion, ^4^ whereas Rab1A exhibits unique functions such as its involvement in cell adhesion and migration, and plays a role in facilitating autophagosome formation, an early step in the autophagy pathway ^7^^,^^8^

Elevated expression of R *AB1A* and *RAB1B* genes have implications in various cancer types, including colorectal cancer, ^9^ hepatocellular cancer, ^10^ gliomas, ^11^ tongue carcinomas, prostate cancer ^12^ for *RAB1A* and colorectal cancer, ^13^ hepatocellular cancer, ^14^ and prostate cancer ^12^ for *RAB1B.* Rab1A in human cancer is highly studied in comparison to Rab1B as abnormal expression of Rab1A activates mTORC1 signalling, promoting tumour growth, invasion and ultimately cancer progression. ^9^ Rab1 proteins are also involved in the pathogenesis of Parkinson’s disease, characterized by accumulation of α-synuclein. Inhibition of ER-Golgi traffic has been reported to trigger α-synuclein aggregation, suggesting that an increase in production of Rab1 proteins can potentially rescue this α-synuclein toxic phenotype. ^15^ Further research is required to understand the role of Rab1A and Rab1B in various diseased states and their potential as therapeutic targets to slow the progression of cancer and neurodegeneration. In-depth mechanistic investigations would significantly benefit from the accessibility of high-performing antibodies, which can help elucidate the underlying processes and pathways involving Rab1A and Rab1B. An editorial by Biddle *et al*. can provide valuable insights on how to interpret the antibody characterization data found in this article. ^16^

This research is part of a broader collaborative initiative in which academics, funders and commercial antibody manufacturers are working together to address antibody reproducibility issues by characterizing commercial antibodies for human proteins using standardized protocols, and openly sharing the data. ^17^^–^^19^ Here, twelve commercially available antibodies that target either Rab1A or Rab1B were tested in Western Blot, immunoprecipitation and immunofluorescence applications using a knockout-based validation approach. This article serves as a valuable guide to help researchers select high-quality antibodies for their specific needs, facilitating the biochemical and cellular assessment of Rab1A and Rab1B properties and function. 

## Results and discussion

Our standard protocol involves comparing readouts from wild-type (WT) and knockout (KO) cells. ^20^^,^^21^ The first step was to identify a cell line(s) that expresses sufficient endogenous levels of a given protein to generate a measurable signal. To this end, we examined the DepMap transcriptomics database to identify all cell lines that express the Rab1 isoforms at levels greater than 2.5 log _2_ (transcripts per million “TPM” + 1), which we have found to be a suitable cut-off (Cancer Dependency Map Portal, RRID:SCR_017655). Commercially available HAP1 cells express Rab1A and Rab1B transcripts at RNA levels above the average range of cancer cells analyzed. Parental and *RAB1A and RAB1B* KO HAP1 cells were obtained from Horizon Discovery ( Table 1).

**Table 1.** Summary of the cell lines used.

Institution	Catalog number	RRID (Cellosaurus)	Cell line	Genotype
Horizon Discovery	C631	CVCL_Y019	HAP1	WT
Horizon Discovery	HZGHC007227c012	CVCL_B5KT	HAP1	*RAB1A* KO
Horizon Discovery	HZGHC001225c011	CVCL_TI00	HAP1	*RAB1B* KO

**Figure 1.**
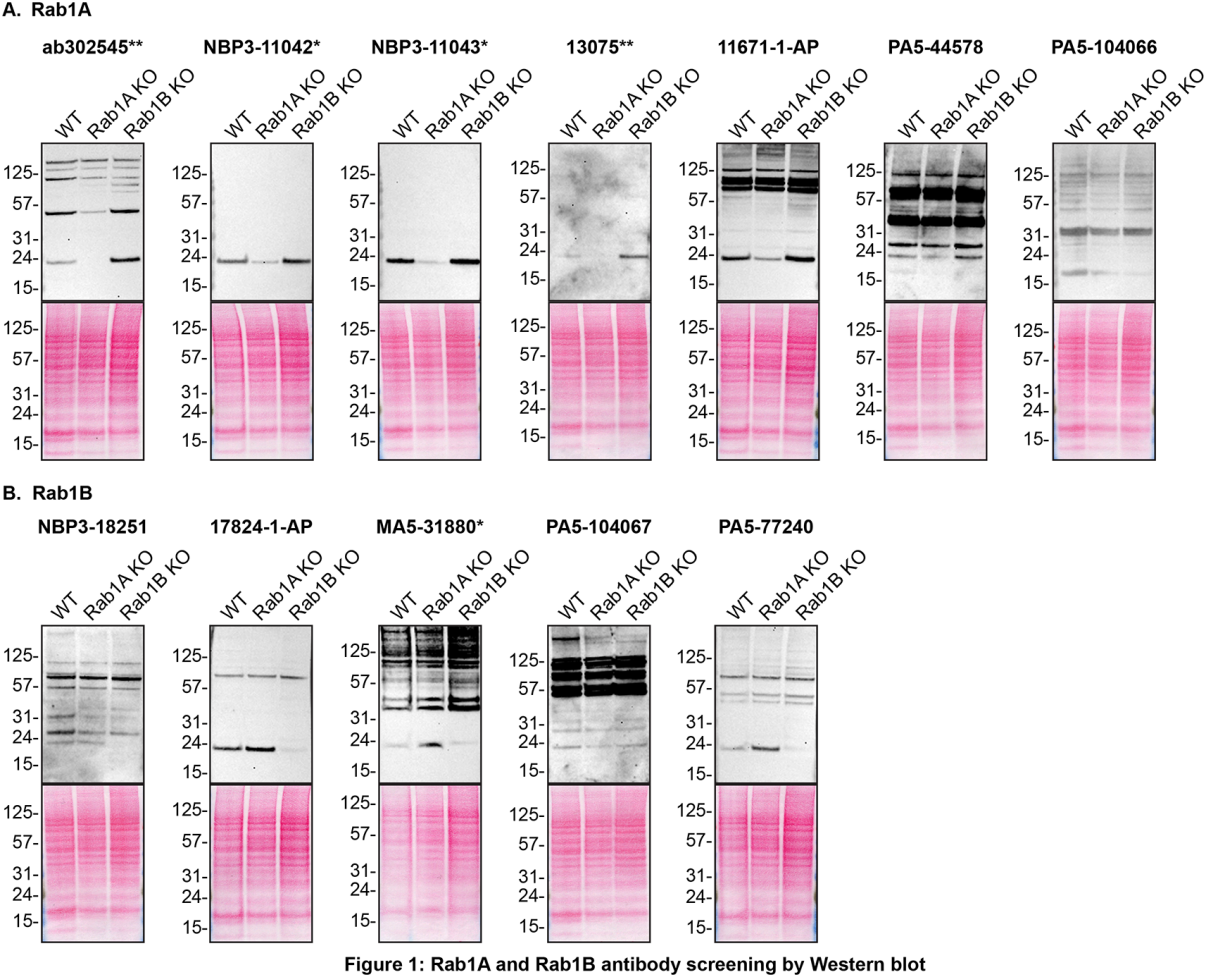
Rab1A and Rab1B antibody screening by Western Blot. Lysates of HAP1 (WT and *RAB1A and RAB1B* KO) were prepared and 30 μg of protein were processed for Western Blot with the indicated Rab1A and Rab1B antibodies. Figure 1A represents the findings from the Western Blot experiments for antibodies intended to bind to Rab1A while Figure 1B represents the Western Blots for antibodies intended to bind Rab1B. The Ponceau stained transfers of each Blot are presented to show equal loading of WT and KO lysates and protein transfer efficiency from the acrylamide gels to the nitrocellulose membrane. Antibody dilutions were chosen according to the recommendations of the antibody supplier. Exceptions were given for antibodies ab302545**, NBP3-11042*, 13075**, 11671-1-AP, PA5-104066, 17824-1-AP, PA5-104067 and PA5-77240, which were titrated to the corresponding dilutions found below, as the signals were too weak when following the supplier’s recommendations. Antibody dilution used: ab302545** at 1/200, NBP3-11042* at 1/500, NBP3-11043* at 1/500, 13075** at 1/200, 11671-1-AP at 1/500, PA5-44578 at 1/500, PA5-104066 at 1/500, NBP3-18251 at 1/200, 17824-1-AP at 1/500, MA5-31880* at 1/500, PA5-104067 at 1/200, PA5-77240 at 1/200. Predicted band size: 22 kDa. *Monoclonal antibody, **Recombinant antibody.

For Western Blot experiments, we resolved proteins from WT and *RAB1A and RAB1B* KO cell extracts and probed them side-by-side with all antibodies in parallel ( Figure 1). ^21^ Figure 1 indicates which antibodies are intended for Rab1A **(A)** or Rab1B **(B)**. In the results, it was observed that Rab1A antibodies, namely ab302545**, NBP3-11042*, NBP3-11043*, 13075** and 11671-1-AP immunodetected their target, Rab1A protein as a ~23 kDa band in the HAP1 WT lysate while the levels of Rab1A increased by ~2-3 fold in the lysates of Rab1B KO cells. Similarly, Rab1B antibodies 17824-1-AP and PA5-77240 detect Rab1B at ~23 kDa in the HAP1 WT lysate, and revealed a similar ~2-3 fold increase in Rab1B protein level in the Rab1A KO lysate. These results demonstrated that a compensatory mechanism exists to ensure that overall Rab1 protein levels remain balanced.

**Figure 2.**
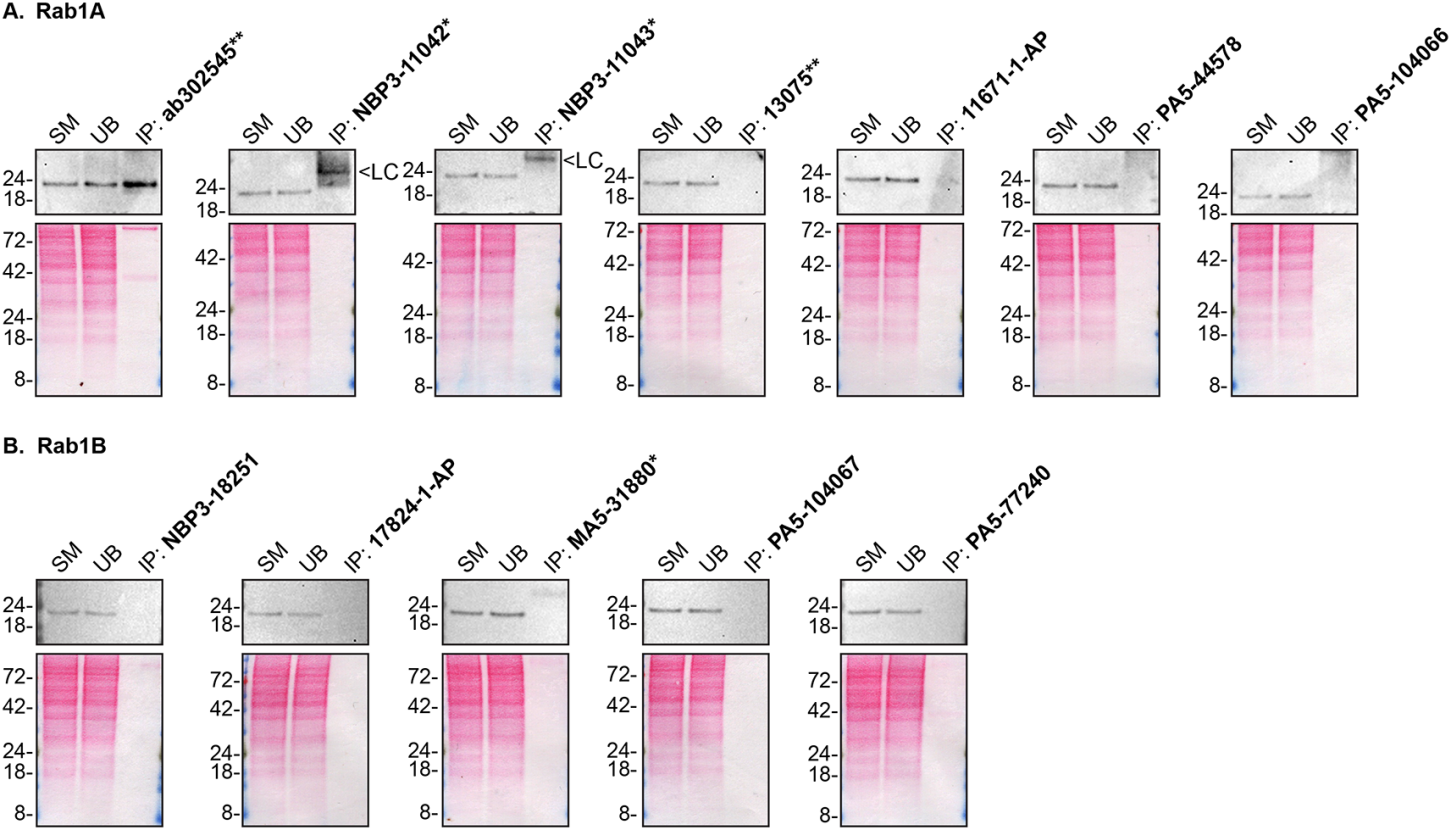
Rab1A and Rab1B antibody screening by immunoprecipitation. HAP1 lysates were prepared, and IP was performed using 2.0 μg of the indicated Rab1A and Rab1B antibodies pre-coupled to Dynabeads protein A or protein G. Samples were washed and processed for Western Blot with the indicated Rab1A and Rab1B antibodies. Figure 2A represents the IP results for antibodies intended to immunodetect Rab1A while Figure 2B represents the results for antibodies intended to immunodetect Rab1B. For Rab1A Western Blots ( **A**), ab302545** was used at 1/200. For Rab1B Western Blots ( **B**), 17824-1-AP was used at 1/500. The Ponceau stained transfers of each Blot are shown. SM=4% starting material; UB=4% unbound fraction; IP=immunoprecipitate; LC=antibody light chain. *Monoclonal antibody, **Recombinant antibody.

As per our standard procedure, we next used the antibodies to immunoprecipitateRab1A and Rab1B from HAP1 cell extracts. The performance of each antibody was evaluated by detecting the Rab1A and Rab1B protein in extracts, in the immunodepleted extracts and in the immunoprecipitates using an antibdy that was validated by Western Blot ( Figure 2). ^21^

**Figure 3.**
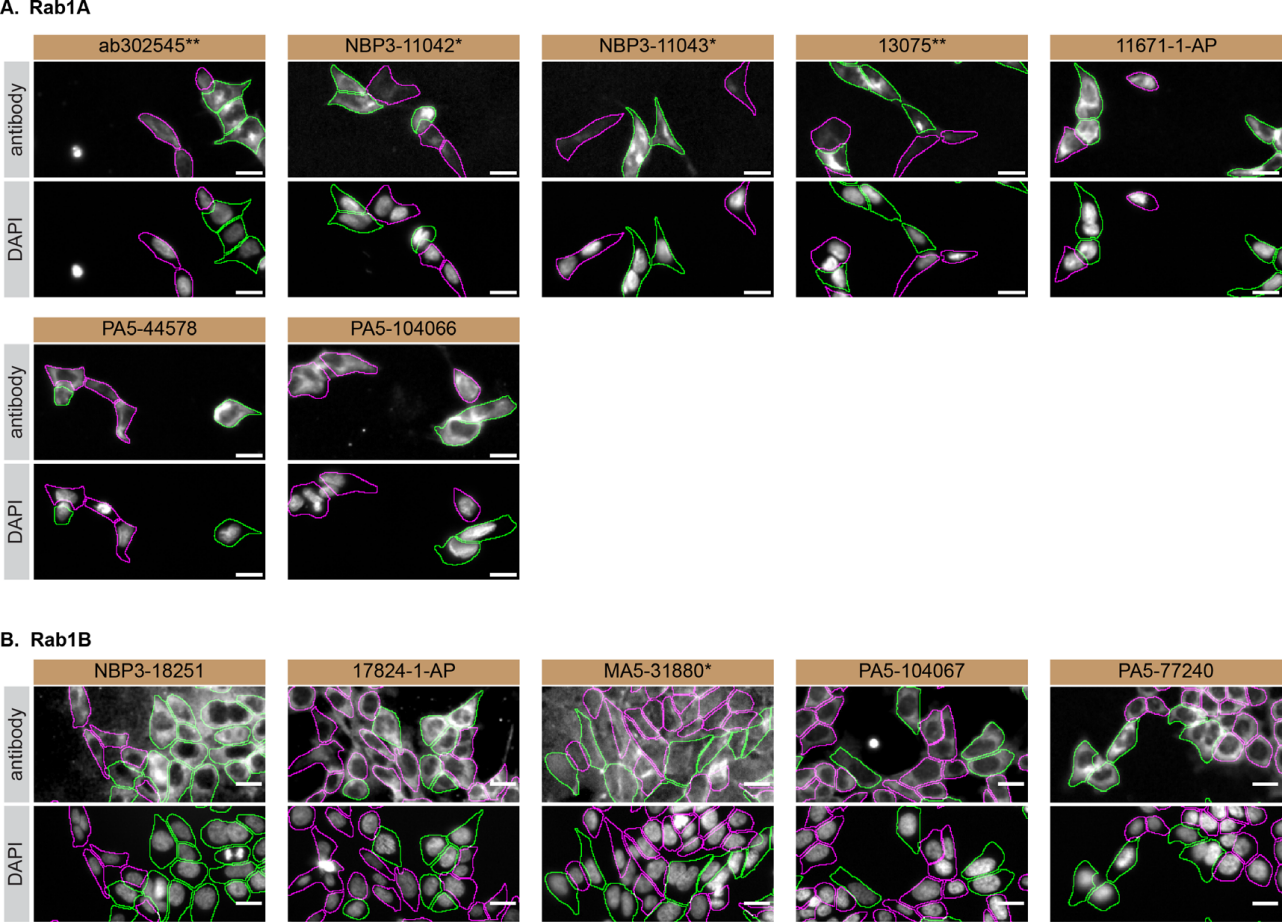
Rab1A and Rab1B antibody screening by immunofluorescence. HAP1 WT were labelled with a green fluorescence dye, and HAP1 *RAB1A* and *RAB1B* KO cells were labelled with a far-red fluorescent dye. Figure 3A represents WT cells plated together *RAB1A* KO, while Figure 3B represents WT cells plated with RAB1B KO cells. WT/KO cells are plated to a 1:1 ratio in a 96-well plate with optically clear flat-bottom. In panel **(A)**, WT and *RAB1A* KO cells were stained with the antibodies intended to target Rab1A, and the corresponding Alexa-fluor 555 coupled secondary antibody including DAPI. In panel **(B)**, WT and *RAB1B* KO cells were stained with the antibodies intended to target Rab1B, and the corresponding Alexa-fluor 555 coupled secondary antibody including DAPI. Acquisition of the blue (nucleus-DAPI), green (identification of WT cells), red (antibody staining) and far-red (identification of KO cells) channels was performed. Representative images of the merged blue and red (grayscale) channels are shown. WT and KO cells are outlined with green and magenta dashed line, respectively. When the concentration was not indicated by the supplier, we tested antibodies at 1/100 or 1/500, which was the case for antibodies NBP3-11042*, NBP3-11043*, PA5-44578, PA5-104066, NBP3-18251 and 17824-1-AP. At these concentrations, the signal from each antibody was in the range of detection of the microscope used. Antibody dilution used: ab302545** at 1/100, NBP3-11042* at 1/100, NBP3-11043* at 1/100, 13075** at 1/800, 11671-1-AP at 1/700, PA5-44578 at 1/500, PA5-104066 at 1/100, NBP3-18251 at 1/100, 17824-1-AP at 1/50, MA5-31880* at 1/1000, PA5-104067 at 1/1000, PA5-77240 at 1/50. Bars = 10 μm. *Monoclonal antibody, **Recombinant antibody.

For immunofluorescence, antibodies were screened using a mosaic strategy, as per our standard procedure. First, the HAP1 WT and *RAB1A* KO cells were plated together in the same tissue culture wells, using different colour fluorescent dyes to distinguish the two cell lines, and the seven Rab1A antibodies were tested. Then, HAP1 WT and *RAB1B* KO cells were plated together using the same strategy, and the five Rab1B antibodies were tested. Cells were imaged in the same field of view to reduce staining, imaging and image analysis bias ( Figure 3). Quantification of immunofluorescence intensity hundreds of WT and KO cells was performedfor each antibody tested. The images presented in Figure 3 are representative of the results of this analysis. 

In conclusion, we have screened seven Rab1A and Rab1B commercial antibodies by Western Blot, immunoprecipitation and immunofluorescence. Several high-quality antibodies that selectively detect either Rab1A or Rab1B under the standardized experimental conditions were identified in each of the tested applications. In our efforts to address the antibody reliability and reproducibility challenges in scientific research, the authors recommend the antibodies that demonstrated to be underperforming be removed from the commercial antibody market. However, the authors do not engage in result analysis or offer explicit antibody recommendations. A limitation of this study is the use of universal protocols - any conclusions remain relevant within the confines of the experimental setup and cell line used in this study. Our primary aim is to deliver top-tier data to the scientific community, grounded in Open Science principles. This empowers experts to interpret the characterization data independently, enabling them to make informed choices regarding the most suitable antibodies for their specific experimental needs.

The underlying data for this study can be found on Zenodo, an open access repository for which YCharOS has its own community. ^22^^,^^23^

## Methods

### Antibodies

All Rab1A and Rab1B antibodies are listed in Table 2, together with their corresponding Research Resource Identifiers, or RRID, to ensure the antibodies are cited properly. ^24^ Peroxidase-conjugated goat anti-rabbit and anti-mouse antibodies are from Thermo Fisher Scientific (cat. number 65-6120 and 62-6520). Alexa-555-conjugated goat anti-rabbit and anti-mouse secondary antibodies are from Thermo Fisher Scientific (cat. number A21429 and A21424).

**Table 2.** Summary of the Rab1A and Rab1B antibodies tested.

Intended Rab-1 target	Company	Catalog number	Lot number	RRID (Antibody Registry)	Clonality	Clone ID	Host	Concentration (μg/μL)	Vendors recommended applications
Rab1A	Abcam	ab302545 **	GR3458765-3	AB_2942108	recombinant-mono	EPR27169-83	rabbit	0.502	WB, IP, IF
Rab1A	Novus Biologicals (a Bio-Techne brand)	NBP3-11042 *	MR288278	AB_2942092	monoclonal	7H4	mouse	1.000	WB, IP
Rab1A	Novus Biologicals (a Bio-Techne brand)	NBP3-11043 *	MR488278	AB_2942109	monoclonal	4G10	mouse	1.000	WB, IP
Rab1A	Cell Signaling Technology	13075 **	1	AB_2665537	recombinant-mono	D3X9S	rabbit	0.200	WB, IF
Rab1A	Proteintech	11671-1-AP	66720	AB_2173437	polyclonal	-	rabbit	0.700	WB, IP, IF
Rab1A	Thermo Fisher Scientific	PA5-44578	YA3805788	AB_2608352	polyclonal	-	rabbit	0.500	WB
Rab1A	Thermo Fisher Scientific	PA5-104066	YA3806218A	AB_2853395	polyclonal	-	rabbit	1.000	WB
Rab1B	Novus Biologicals (a Bio-Techne brand)	NBP3-18251	PR285216	AB_2942110	polyclonal	-	rabbit	1.000	WB, IF
Rab1B	Proteintech	17824-1-AP	39221	AB_2237881	polyclonal	-	rabbit	0.200	WB, IF
Rab1B	Thermo Fisher Scientific	MA5-31880 *	YA3806197	AB_2787503	monoclonal	7A12G2	mouse	1.000	WB
Rab1B	Thermo Fisher Scientific	PA5-104067	YA3806219A	AB_2853396	polyclonal	-	rabbit	1.000	WB, IF
Rab1B	Thermo Fisher Scientific	PA5-77240	YA3805998	AB_2720967	polyclonal	-	rabbit	1.000	WB, IF

WB=Western Blot; IF=immunofluorescence; IP=immunoprecipitation.

* Monoclonal antibody,

** Recombinant antibody

### Cell culture

Both HAP1 WT and *RAB1A and RAB1B* KO cell lines used are listed in Table 1, together with their corresponding RRID, to ensure the cell lines are cited properly. ^25^ Cells were cultured in DMEM high-glucose (GE Healthcare cat. number SH30081.01) containing 10% fetal bovine serum (Wisent, cat. number 080450), 2 mM L-glutamate (Wisent cat. number 609065), 100 IU penicillin and 100 μg/mL streptomycin (Wisent cat. number 450201).

### Antibody screening by Western Blot

Western Blots were performed as described in our standard operating procedure. HAP1 WT and the HAP1 *RAB1A and RAB1B* KO lines (listed in Table 1) were collected in RIPA buffer (25 mM Tris-HCl pH 7.6, 150 mM NaCl, 1% NP-40, 1% sodium deoxycholate, 0.1% SDS) from Thermo Fisher Scientific (cat. number 89901) supplemented with 1× protease inhibitor cocktail mix (MilliporeSigma, cat. number P8340). Lysates were sonicated briefly and incubated for 30 min on ice. Lysates were spun at ~110,000 × g for 15 min at 4°C and equal protein aliquots of the supernatants were analyzed by SDS-PAGE and Western Blot. BLUelf prestained protein ladder from GeneDireX (cat. number PM008-0500) was used.

Western Blots were performed with a precast midi 10% Bis-Tris polyacrylamide gels from Thermo Fisher Scientific (cat. number WG1201BOX) ran with MES SDS buffer (Thermo Fisher Scientific, cat. number NP000202), loaded in LDS sample buffer (Thermo Fisher Scientific, cat. number NP0008) with 1× sample reducing agent (Thermo Fisher Scientific, cat. number NP0009) and transferred on nitrocellulose membranes. Proteins on the Blots were visualized with Ponceau S staining (Thermo Fisher Scientific, cat. number BP103-10) which is scanned to show together with individual Western Blot. Blots were blocked with 5% milk for 1 hr, and antibodies were incubated overnight at 4°C with 5% milk in TBS with 0.1% Tween 20 (TBST) (Cell Signalling Technology, cat. number 9997). Following three washes with TBST, the peroxidase conjugated secondary antibody was incubated at a dilution of ~0.2 μg/mL in TBST with 5% milk for 1 hr at room temperature followed by three washes with TBST. Membranes were incubated with Pierce ECL from Thermo Fisher Scientific (cat. number 32106) or Clarity Western ECL Substrate from Bio-Rad (cat. number 1705061) prior to detection with the iBright™ CL1500 Imaging System from Thermo Fisher Scientific (cat. number A44240). Membranes incubated with primary antibodies NBP3-11043*, 13075**, PA5-44578, PA5-104066, NBP3-18251 and PA5-104067 were developed with Clarity Western ECL Substrate, and the remaining antibodies with Pierce ECL.

### Antibody screening by immunoprecipitation

Immunoprecipitation was performed as described in our standard operating procedure. Antibody-bead conjugates were prepared by adding 2 μg or 10 μL of antibody NBP3-18251 (unknown concentration) to 500 μL of Pierce IP Lysis Buffer from Thermo Fisher Scientific (cat. number 87788) in a 1.5 mL microcentrifuge tube, together with 30 μL of Dynabeads protein A - (for rabbit antibodies) or protein G - (for mouse antibodies) from Thermo Fisher Scientific (cat. number 10002D and 10004D, respectively). Tubes were rocked for ~1 hr at 4°C followed by two washes to remove unbound antibodies.

HAP1 WT were collected in Pierce IP buffer (25 mM Tris-HCl pH 7.4, 150 mM NaCl, 1 mM EDTA, 1% NP-40 and 5% glycerol) supplemented with protease inhibitor. Lysates were rocked for 30 min at 4°C and spun at 110,000 × g for 15 min at 4°C. 0.5 mL aliquots at 2.0 mg/mL of lysate were incubated with an antibody-bead conjugate for ~1 hr at 4°C. The unbound fractions were collected, and beads were subsequently washed three times with 1.0 mL of IP lysis buffer and processed for SDS-PAGE and Western Blot on a precast midi 10% Bis-Tris polyacrylamide gels. Prot-A: HRP (MilliporeSigma, cat. number P8651) was used as a secondary detection system at a concentration of 0.3 μg/mL.

### Antibody screening by immunofluorescence

Immunofluorescence was performed as described in our standard operating procedure. ^21^ HAP1 WT and the HAP1 *RAB1A and RAB1B* KO cell lines were labelled with a green and a far-red fluorescence dye, respectively. The fluorescent dyes used are from Thermo Fisher Scientific (cat. number C2925 and C34565). The nuclei were labelled with DAPI (Thermo Fisher Scientific, cat. number D3571) fluorescent stain. WT and KO cells were plated in a 96-well plate with optically clear flat-bottom (Perkin Elmer, cat. number 6055300) as a mosaic and incubated for 24 hrs in a cell culture incubator at 37°C, 5% CO _2_. Cells were fixed in 4% paraformaldehyde (PFA) (Beantown chemical, cat. number 140770-10 ml) in phosphate buffered saline (PBS) (Wisent, cat. number 311-010-CL) for 15 min at room temperature and washed 3 times with PBS. Cells were permeabilized in PBS with 0.1% Triton X-100 (Thermo Fisher Scientific, cat. number BP151-500) for 10 min at room temperature and blocked with PBS with 5% bovine serum albumin (BSA) (Wisent, cat. number 800-095), 5% goat serum (Gibco, cat. number 16210-064) and 0.01% Triton X-100 for 30 min at room temperature. Cells were incubated with IF buffer (PBS, 5% BSA, 0.01% Triton X-100) containing the primary Rab1A and Rab1B antibodies overnight at 4°C. Cells were then washed 3 × 10 min each with IF buffer and incubated with corresponding Alexa Fluor 555-conjugated secondary antibodies in IF buffer at a dilution of 1.0 μg/mL for 1 hr at room temperature with DAPI. Cells were washed 3 × 10 min with IF buffer and once with PBS.

Images were acquired on an ImageXpress micro widefield high-content microscopy system (Molecular Devices), using a 20x NA 0.95 water objective lens and scientific CMOS camera (16- bit, 1.97 mm field of view), equipped with 395, 475, 555 and 635 nm solid state LED lights (Lumencor Aura III light engine) and bandpass emission filters (432/36 nm, 520/35 nm, 600/37 nm and 692/40 nm) to excite and capture fluorescence emission for DAPI, CellTracker ^TM^ Green, Alexa fluor 555 and CellTracker ^TM^ Red, respectively. Images had pixel sizes of 0.68 × 0.68 microns. Exposure time was set with maximal (relevant) pixel intensity ~80% of dynamic range and verified on multiple wells before acquisition. Since the IF staining varied depending on the primary antibody used, the exposure time was set using the most intensely stained well as reference. Frequently, the focal plane varied slightly within a single field of view. To remedy this issue, a stack of three images per channel was acquired at a z-interval of 4 microns per field and best focus projections were generated during the acquisition ( MetaXpress v6.7.1, Molecular Devices). Segmentation was carried out on the projections of CellTracker ^TM^ channels using CellPose v1.0 on green (WT) and far-red (KO) channels, using as parameters the ‘cyto’ model to detect whole cells, and using an estimated diameter tested for each cell type, between 15 and 20 microns. ^26^ Masks were used to generate cell outlines for intensity quantification. Figures were assembled with Adobe Photoshop (version 24.1.2) to adjust contrast then assembled with Adobe Illustrator (version 27.3.1).

## Data Availability

Zenodo: Antibody Characterization Report for Rab1A and Rab1B, https://doi.org/10.5281/zenodo.8356353. ^22^ Zenodo: Dataset for the Rab1A and Rab1B antibody screening study, https://doi.org/10.5281/zenodo.8400619. ^23^ Data are available under the terms of the Creative Commons Attribution 4.0 International license (CC-BY 4.0)
