# Supplementary material for: A guide to selecting high-performing antibodies for Rab1A and Rab1B for use in Western Blot, immunoprecipitation and immunofluorescence
Source: F1000Res. 2025 Mar 7;12:1578. Originally published 2023 Dec 11. [Version 3] doi: 10.12688/f1000research.143928.3 (PMC10979127; doi:10.12688/f1000research.143928.3)
Supplement: Peer review history

# Reviewer report: Reviewer response for version 2

**DOI:** 10.5256/f1000research.160069.r253285

**Published:** 2024-03-28

**Authors:** Claudia Fallini

**Affiliations:** 1 University of Rhode Island, Kingston, Rhode Island, USA

- In this report, Moleon and colleagues characterize several antibodies directed against the human Rab1A or Rab1B proteins. Rab1 proteins are small GTP-binding proteins that participate in vesicle trafficking, among other cellular functions. Interest in these proteins also stems from their potential role as oncogenes, as several cancer types have been shown to upregulate Rab1A or Rab1B. The authors carefully characterized 7 Rab1A and 5 Rab1B antibodies by western blot, immunoprecipitation, and immunofluorescence assays using KO and isogenic parental cell lines. The results are clearly presented, and the methods detailed enough to allow replication. Overall, there are no specific concerns in my view.
- Only few minor typos were identified such as missing spaces in the abstract ("diseaseand") and on page 5 ("immunoprecipitateRab1A", "antibdy", and "performedfor").

Are sufficient details of methods and materials provided to allow replication by others?

Yes

Is the rationale for creating the dataset(s) clearly described?

Yes

Are the datasets clearly presented in a useable and accessible format?

Yes

Are the protocols appropriate and is the work technically sound?

Yes

Reviewer Expertise:

Cell biology and neuroscience

I confirm that I have read this submission and believe that I have an appropriate level of expertise to confirm that it is of an acceptable scientific standard.

# Reviewer report: Reviewer response for version 2

**DOI:** 10.5256/f1000research.160069.r246962

**Published:** 2024-02-26

**Authors:** Shinsuke Niwa

**Affiliations:** 1 Tohoku University, Miyagi, Japan

1. It would be helpful to have a table describing which antibodies are useful and reliable and which are not for each application. 

 2. A limitation of this study is that the authors analyzed only the human cell line HAP1 and not widely used cells such as Hela cells and 293 cells.

 3. While "the authors recommend the antibodies that demonstrated to be underperforming be removed from the commercial antibody market", one cannot conclude usefulness of these antibodies in cell lines derived from other species such as mouse, hamster (CHO cells) and rat.

Are sufficient details of methods and materials provided to allow replication by others?

Yes

Is the rationale for creating the dataset(s) clearly described?

Partly

Are the datasets clearly presented in a useable and accessible format?

Yes

Are the protocols appropriate and is the work technically sound?

Yes

Reviewer Expertise:

cell biology

I confirm that I have read this submission and believe that I have an appropriate level of expertise to confirm that it is of an acceptable scientific standard.
